# On Some of the Medicinal Properties and Therapeutical Applications of Chlorate of Potassa

**Published:** 1857-05

**Authors:** J. S. Weatherly

**Affiliations:** Montgomery, Ala.


### ARTICLE II.

*On some of the Medicinal Properties and Therapeutical Applications of Chlorate of Potassa.* By J. S. WEATHERLY, M. D., Montgomery, Ala.

In compliance with a long neglected promise, of furnishing an occasional article for the columns of your Journal, I proceed to offer a few wandering thoughts upon an article of the Materia Medica, whose merits, as a remedial agent, I am inclined to think are not properly appreciated by the profession. I mean the Potassæ Chloras. Its name carrys with it the chemical constituents of the substance. The chemical composition of this article would leave us to ascribe alterative virtues to it—such as are claimed for it by those who have tested its virtues. In offering a few remarks upon this article, I do not expect to startle the medical world with any thing new or original, neither with the idea that I am advocating a specific for any disease. Neither do I wish to lead any one into error, by presenting undigested theories for their consideration. For the profession is already overshadowed by crude theories and fancies, emanating from the brains of those whose patients happen to get well, with or without the influence of a certain remedy, when, forthwith is heralded to the medical world, a specific. But, lo! when the vaunted specific comes to be tried by others, we hear muttered anathemas, and curses long and deep from the disappointed ones, against the unfortunate discoverer of a specific. Hence, it is, that much, very much, that is written for Medical Journals is doomed to pass unread, or to be consigned to the "tomb of the capulets," as being unworthy of remembrance. It may be so with this article. And, if so, I say, "so mote it be;" for I, for one, am anxious to see medical facts advanced, and sustained by philosophic reason, and confirmed by practical experience. My attention was first particularly attracted to this article by an essay, by Professor Frost, of Charleston, in the Charleston Medical Journal, several years ago. He recommended it for continued and remittent fevers. Upon a farther examination, I found it highly extolled by several German and English authors of high reputation. Numerous theories were advanced to account for its actions, but as I have no desire to engage in vague speculations, and no particular theory to advocate, I shall leave this part of the subject for minds better adapted to hypothesies than my own. I have used the remedy, and have thought I derived advantage from its use. But as the class of diseases, for which I have generally employed it, are not such, as yield positive results to any remedy, it is difficult for me to say how much benefit I have derived from its use. The diseases for which I have employed this remedy, with apparent good effects, are Typhoid Fever, Scarlatina and Croup, and in all diseases attended with a low form of inflammation and ulceration of the mucus membrane of the intestinal canal.

I have used it in all stages of typhoid fever, generally with the view of its action upon the mucus membrane of the intestine. How it prevents ulceration, or cures it when it occurs, I am not prepared to say, but that it will do it, I think amounts almost to a certainty. Any one who has witnessed its prompt action in arresting ulceration of the mouth and fauces, would would naturally infer, that it would have the same effect upon the same membrane in a different situation. It follows then, that the indications for its use in fevers, are such as show a tendency to inflammation and ulceration, where the tongue is dry and red, with sordes upon the teeth, a quick and feeble pulse, where an alterative of some kind is necessary, it may generally be used with benefit.

In Scarlatina, especially, the malignant variety attended with ulceration and gangrene of the fauces, it is a remedy not only internally, but as a gargle. Besides its action upon the inflamed mucus membrane, it also acts as an alterative to the blood. How it acts as a depurating agent upon the blood, I am not prepared to say. But that it is especially adapted to diseases of blood poisoning, I think I am warranted in saying, from experience. It may be by supplying oxygen, or the chlorine, may act as a purifying agent as it does out of the system. I will here give a case of croup which was treated by me several years since, which will illustrate its effect upon deficiently arterialised blood. The child had been suffering from croup for two or three days previous to my seeing it, and had been under the influence of nauseants and emetics all the time. When I saw it, it was gasping for breath, the face and lips blue and torpid; the whole surface cold and clammy; pulse scarcely perceptible; in fact, all the symptoms indicating a speedy termination of life. Not being prepared to perform tracheotomy—for I really thought that the only chance of life—I determined to try the chlorate as a dernier resort; thinking that I might probably act on the blood with it, and also counteract the peculiar condition of the mucus membrane of the larynx and trachea. I commenced with one teaspoonful of the saturated solution every hour; and very much to my surprise, and to the gratification of the parents, the child was convalescent in about four hours. It may have been chance, but if it was, when and how are we to determine when a remedy does act beneficially? A few words as to its administration and I am done. I usually make a saturated solution of the chlorate in aqua pura, and administer one tablespoonful to an adult every two or three hours, according to circumstances.

I will now conclude this hasty article. I have given it to you in my own words, and with my own ideas, not having the leisure or desire to indulge in long or numerous quotations for the purpose of filling out my paper.

